# Different hydraulic and photosynthetic responses to summer drought between newly sprouted and established Moso bamboo culms

**DOI:** 10.3389/fpls.2023.1252862

**Published:** 2023-10-12

**Authors:** Xin Zhang, Chazi Tong, Dongming Fang, Tingting Mei, Yan Li

**Affiliations:** ^1^ State Key Laboratory of Subtropical Silvilculture, Zhejiang A&F University, Lin’an, Zhejiang, China; ^2^ College of Horticulture, Jiyang College of Zhejiang Agriculture and Forestry University, Zhuji, Zhejiang, China

**Keywords:** drought stress, tree mortality, Moso bamboo, age effect, hydraulic failure

## Abstract

The subtropical regions in China are prone to recurrent summer droughts induced by the Western Pacific Subtropical High-Pressure, which has induced the death of tens of millions of culms of Moso bamboo (*Phyllostachys edulis* (Carriere) J. Houzeau), a widely distributed giant bamboo with high economic and ecological values. In the future, the intensity and frequency of the summer drought are projected to increase in these areas due to global climate change, which may lead to significant age-specific mortality of Moso bamboo. So far, it is still unclear about the age-specific response mechanisms of hydraulic traits and carbon balance of Moso bamboo when it is suffering to an ongoing summer drought. This study aimed to investigate the hydraulic and photosynthetic responses of newly sprouted (1 year old) and established (2-5 years old) culms of Moso bamboo to summer drought, which was manipulated by throughfall reduction in Lin’an of Zhejiang. The results showed that both newly sprouted and established culms had a gradually weakening hydraulic conductivity and photosynthesis during the whole drought process. In the early stage of the manipulated drought, the established culms had more loss of hydraulic conductivity than the newly sprouted culms. However, the newly sprouted culms had significant more loss of hydraulic conductivity and lower photosynthetic rates and stomatal conductance in the middle and late stages of the manipulated drought. The results suggest that the newly sprouted culms were more susceptible to summer drought than established culms due to the combined effects of hydraulic damage and photosynthetic restriction, explaining why the newly sprouted culms have higher mortality than elder culms when subjected to extreme drought. These findings provided insights into the mechanisms of Moso bamboo’s age-specific drought-induced mortality, which will help for the anti-drought management of bamboo.

## 1 Background

The impact of droughts, coupled with high temperatures, has posed significant challenges to forest ecosystems (Zhao and Running, 2010; IPCC, 2018; FAO, 2019; IPCC, 2022), causing extensive tree mortality (Adams et al., 2010; Allen et al., 2010; Klein and Hartmann, 2018; Powers et al., 2020; Senf et al., 2020). Severe tree mortality can potentially change the composition of species in the impacted forest ecosystem (Ruiz-Benito et al., 2017; Gazol et al., 2018), and this shift may depend on the drought response of the standing trees and saplings (Phillips et al., 2010; Lindenmayer et al., 2012; Lutz et al., 2012). While some studies found that adult trees were more vulnerable to drought due to higher evaporative demand (Phillips et al., 2010; Lindenmayer et al., 2012; Lutz et al., 2012), others suggested that saplings were more prone to mortality because of narrower root systems (Padilla and Pugnaire, 2007; Lloret et al., 2009). Despite the significant loss of carbon and ecosystem function that results from adult tree mortality, greater sapling mortality may alter future forest succession patterns under climate change and possible drought threats (Phillips et al., 2010; Lindenmayer et al., 2012; Lutz et al., 2012). Given the potential consequences of tree mortality, it is crucial to monitor and assess the age-specific performance of component species in forests, particularly those that are dominant or constructive in regions that may experience drought in the future (Martínez-Vilalta and Lloret, 2016). This information is essential for forecasting the future structure and composition of forest ecosystems.

Moso bamboo (*Phyllostachys edulis* (Carriere) J. Houzeau) is a fast-growing species that has a high carbon sequestration capacity and explosive growth rate (Zhou and Jiang, 2004; Song et al., 2017). It covers more than 4.67 million hectares of forest land in China, mainly in the subtropical area (National Forestry and Grassland Administration, 2019). However, the subtropical regions in China are prone to recurrent summer droughts, characterized by elevated temperatures and reduced precipitation, which are induced by the influence of the Western Pacific Subtropical High-Pressure system (Tong et al., 2021). These droughts are likely to worsen due to global climate change and may increase the mortality of the dominant plants in the region (Chai et al., 2019, Xu et al., 2018). If the prolonged heat and dryness continue, the survival of the bamboo forests in this area will be at risk as the species adapted to moist climates may not cope well with extreme drought stress and die (Wang and Wang, 2023). Extreme drought events may have more negative effects on such plants than gradual environmental changes, as they do not allow enough time for the plants to adjust to the drought conditions (Jentsch et al., 2007), as observed in some tropical rain forests (O’Brien et al., 2017). In recent years, a comparable situation also happened to Moso bamboo forest in the subtropical area of China; for example, a severe summer drought that lasted for 40 days in 2013 killed tens of millions of Moso bamboo (Liu et al., 2014).

Recent studies indicated that Moso bamboo culms at different developmental stages had diverse water use, forming a decreasing trend with age (Zhao et al., 2017; Gu et al., 2019; Tong et al., 2021). They observed a decline in both sap flow density and whole-tree hydraulic conductance as the culm age from juvenile to senescent. Such a decreasing trend is thought to relate to enlargement of xylem embolism in aging culms, as Moso bamboo cannot renew the xylem by enlarging their culms (Liese, 1998; Tong et al., 2021). Therefore, elder culms of Moso bamboo may tend to avoid drought-induced death by quicker adjustment than young culms, e.g., closing leaf stomata and reducing transpiration. In the previous throughfall-excluded experiments (Wu et al., 2019; Tong et al., 2021), the researchers found that, compared with elder culms, young culms adopted more risky drought-tolerant strategy to response to summer severe drought (Tong et al., 2021) or longer-term moderate drought (Wu et al., 2019), i.e., maintaining high transpiration for a longer period. Such a water use strategy induced a lower leaf water potential of the young culms (Wu et al., 2019), which was supposed to increase the risk of water transport system and even more severe consequences-hydraulic failure and death. The assumption can be indirectly reflected by the higher mortality percentage (> 70%) of newly sprouted culms during the post-drought investigation (Liu et al., 2014). However, few studies have reported on the age-specific drought-induced mortality mechanisms of Moso bamboo during periods of summer drought.

In recent decades, various studies and theories have been put forward to explain the mechanisms behind tree mortality caused by drought. These encompass hydraulic failure and carbon starvation (McDowell, 2011; Adams et al., 2017), with much research suggesting that hydraulic failure is the primary factor (Anderegg et al., 2016). The hydraulic failure hypothesis indicates that drought-induced soil drying can result in xylem cavitation and embolization, which impedes water transport and ultimately causes cellular death when internal water reserves are depleted (Sperry and Love, 2015; Körner, 2019). In addition, as drought persists, the closure of leaf stomata reduces plant photosynthesis, leading to a decrease in carbon assimilation (Pangle et al., 2015; Tomasella et al., 2019). However, long-term data on the physiological response of trees to drought are scarce (Breshears et al., 2009). Instead, experiments that manipulate rainfall and include appropriate controls can provide valuable insights. A manipulated throughfall exclusion experiment in the plot can achieve severe environment by simulating flash drought in summer, thereby more accurately and quickly exploring the mechanism of plant mortality caused by drought.

In this study, to investigate the response mechanisms of Moso bamboo to summer drought, a manipulated throughfall reduction experiment was conducted in a Moso bamboo forest. Our research centered on alterations in water utilization of different organs, as well as changes in foliar stomatal apertures in bamboo culms at different developmental stages. We hypothesized that the hydraulic response of Moso bamboo to summer drought was a holistic response rather than functional changes of a single organ. In addition, we proposed that the newly sprouted culms may be more susceptible to summer drought than established culms. Our objectives were to explore: 1) how the hydraulic traits of Moso bamboo change in response to summer drought; 2) the impacts of severe manipulated drought on the physiological properties of Moso bamboo at different developmental stages.

## 2 Materials and methods

### 2.1 Study sites

The study was conducted in a bamboo forest in Lin’an (30°14′N, 119°42′E), Zhejiang Province, China. The area falls within the subtropical monsoon climate zone and experiences an average annual precipitation of 1522 ± 518.8 mm and a mean annual temperature of 17.0 ± 2.6°C, characterized by elevated temperatures (27.5 vs. 24.8°C in June) and reduced precipitation (159.8 vs. 290.7 mm in June) in summer-induced by the Western Pacific Subtropical High-Pressure system (Tong et al., 2021). The studied Moso bamboo stand is on the north slope of a low hill with a slope gradient of 36.5°, and the soil type in the study site is ferrisols derived from granite. The stand density is 2150 culms ha^-1^, and the newly sprouted (1 year old) and established (2-5 years old) account for nearly 15% and 85% of the total culms. The studied bamboo forest is almost natural without additional management.

### 2.2 Experimental drought manipulation and soil moisture measurement

In 2019, two permanent plots were established, each measuring 18 m × 36 m, and were subjected to throughfall reduction and control treatments, respectively. To achieve throughfall reduction, polyolefin film roofs were installed on rails at a height of approximately 2 m above the ground to collect and drain rainwater from the plot (**Appendix Figures 1**, **2**). The control plot did not have any plastic roofs. To stop the movement of water sideways and eliminate any clonal integration from bamboo outside the plot, we dug a trench at 50 cm depth around the plots.

The experimental subjects for each plot were 12 pairs of newly sprouted culms (1 year old) and established culms (2-5 years old), about 14 m in height and with the rhizome connected to them. To minimize the impact of other culms via rhizomes, the other culms were cut. During the drought-progressing stage, i.e., early, middle, and late stages from 14 - 31 August, 1 - 15 September, and 16 - 29 September 2019, respectively, three pairs of culms were measured on three sunny days (17-19 August, 9-11 September, and 27-29 September).

In each plot, three time-domain reflectometry sensors (CSC616, Campbell Scientific, USA) were inserted vertically into the ground to measure soil moisture (SM, m^3^·m^-3^) at the top 20 cm layer of soil. Data were collected and recorded by dataloggers (CR1000, Campbell Scientific, USA) at 10-minute intervals. Field water capacity (FWC, %) was determined using a circular soil cutter and the gravimetric method (Reynolds, 1970). Finally, relative water content of soil (RWC, %) was calculated using the following equation:


(1) RWC = SM / FWC        

### 2.3 Leaf water potential and leaf hydraulic conductivity

The *in-situ* leaf water potential (*Ψ*, MPa) of studied culms was measured every two hours between 06:00 and 18:00 on three continuous cloudless days using a Microvoltometer and an L-51 Leaf Hygrometer/Psychrometer (PSYPRO, WESCOR, USA). Three leaves (sun-exposed) per culm were collected for the measurement.

To obtain hydraulic conductivity of leaves, branches, and culms at three stages of the drought manipulation period, i.e., early, middle, and late stages, three bamboo culms pairs in each plot were harvested in the early morning (6:00 - 7:00) of a sunny day in each stage. The selected bamboo culms at the base were cut into one-meter lengths and the surround of two ends were wrapped with degreasing cotton soaked in 20 mmol·L^-1^ KCl solution. Branches with leaves were collected and soaked in the KCl solution and brought back to the laboratory as soon as possible.

Two essential variables, i.e., leaf capacitance (*C*_leaf-area_, mmol·m^-2^·MPa^-1^) and leaf water potential (*Ψ*, MPa) should be obtained for calculating leaf hydraulic conductivity (*K*_L_, kg·m·s^-1^·MPa^-1^) according to the following equation (Brodribb and Holbrook, 2004; Yang et al., 2012):


(2) $$K_{\text{L}}\;=\;C_{leaf-area}\;\times \text{ ln}(\Psi_{\text{i}}\;/\;\Psi_{\text{s}}\;)\;/\;t$$

Where *Ψ*_i_ is the initial leaf water potential (MPa), *Ψ*_s_ is the leaf water potential in water saturation condition (MPa), *t* is the rehydration time which was set to 120 s in this study (s). Small branches were cut in the morning and sealed into moist black plastic bags for 30 minutes to assure the whole leaves had homogeneous water potential and stomata were closed. After measuring *Ψ*_i_ of the selected branch, the leaves were harvested underwater and allowed to absorb water for 120 s so that *Ψ*_s_ would be approximately half of *Ψ*_i_.

To obtain leaf capacitance (Yang et al., 2012), leaves were soaked in KCl solution for about one hour to get water saturated and then surface was dried with tissues. Leaves were weighed to get a fresh weight (*m*_0_, kg) and then measured for the first leaf water potential (*Ψ*_0_, MPa). Leaf area (*A*_L_, m^2^) was assessed using a portable leaf area meter (AM-300, LI-COR Inc., USA). After the first measurement, leaves were dried in room temperature and re-weighed to obtain weights (*m*_f_, kg) in every one minute and the corresponding water potential (*Ψ*_f_, MPa) until *Ψ*_f_ reach its minimum value. Finally, measured leaves were dried under 70 °C to get a constant dry weight (*m*_d_, kg). The relative water content (RWC_f_) of leaves was calculated as


(3) $${\text{RWC}}_{\text{f}}\;=\;(m_{\text{f}}\;-\;m_{\text{d}})\;/\;(m_{0}\;-\;m_{\text{d}})\;\times \;100$$

A linear relationship between 1-RWC (*Y*) and -1/*Ψ* (*X*) was built for each leaf with the following equation:


(4) Y = k × X + b

Where *k* and *b* is parameters for each leaf derived from equation (4).

Leaf capacitance was derived with the following equation (Tyree and Sperry, 1989; Yang et al., 2012):


(5) $$C_{\text{leaf}-\text{area}}\;=\;k\;\times \;\left(m_{d}/A_{\text{L}}\right)\;\times \left(m_{0}\;/m_{\text{d}}\right)/\;M$$

Where *k* is derived from equation (4), *m*_d_ is the constant dry weight of leaf (kg), *m*_0_ is the initial fresh weight of leaf (kg), *A*_L_ is the leaf area (m^2^), *M* is the molar mass of water (g·mol^-1^).

To obtain the maximum hydraulic conductivity *K*_L-max_ (kg·m·s^-1^·MPa^-1^), a relationship between *K*_L_ and *Ψ*_s_ was built for each tested leaf according to equation (1). The *K*_max_ was derived when *Ψ*_i_ set to 0 (Brodribb and Holbrook, 2003; Brodribb et al., 2010). The Percentage Loss of Conductivity (PLC, %) was derived with the following equation:


(6) $$\text{PLC }=\;\left(1\;-\;K_{\text{L}}\;/\;K_{\text{L}-\text{max}}\right)\;\times \;100$$

The specific hydraulic conductivity (*K*_L-s_, kg·m^-1^·s^-1^·MPa^-1^) was derived with the following equation:


(7) $$K_{\text{L}-\text{s}}\;=\;K_{\text{L}}\;/\;A_{\text{L}}$$

### 2.4 Hydraulic conductivity of branches and culms

The hydraulic conductivity of branches and culms was determined with the method referring to Yang et al. (2012). In the lab, the branches and culm segment were re-cut under water to get a 20 cm long segment (*L*, m). The length of the segment was determined in a pre-experiment with the air method (Zimmermann and Jeje, 1981). Then, two ends of the segment were polished, diameters were measured, and cross-sectional areas (*A*_s_, m^2^) were determined. A water reservoir, filled with 20 mmol·L^-1^ KCl solution, was attached to the upper end of the culm through a connector and a plastic tube, and was placed 1 m height above the segment to obtain approx. 0.01 MPa of water pressure. The segment was placed horizontally on a desk and the lower end was set over the collecting cup sat on a balance. After a short equilibration period, water flow (*F*, kg·s^-1^) through the segment were determined by measuring weight of water in a given period. Conductivity of branches and culms (*K*_B/C_, kg·m·s^-1^·MPa^-1^) was determined according to the following equation:


(8) $$K_{\text{B}/\text{C}}=\;F\;\times \;L\;/\text{ Δ}P\;=\;F\;\times \;L\;/\;0.01$$

After the initial hydraulic conductivity was measured, the branches and culms were washed with the KCl solution under a pressure of 0.15 - 0.2 MPa until no bubbles were observed from the end of the segment. Then the maximum hydraulic conductivity (*K*_B/C-max_, kg·m·s^-1^·MPa^-1^) was obtained (Tyree and Sperry, 1989; Brodribb et al., 2010; Yang et al., 2012). PLC of branches and culms and *K*_B/C-s_ were obtained according to equations (6) and (7).

### 2.5 Photosynthetic traits measurements

To explore characteristics of leaf photosynthetic light-response curve over drought processes, three pairs of the newly sprouted-established bamboos were selected in both the control and the throughfall-reduction plots. Bamboo ladders were built to reach the canopy of the studied bamboo culms. Light-response curve of leaf photosynthesis was measured with Li-Cor 6400 (LI-6400XT, LI-COR, USA) on three sun-exposed leaves for each studied culm. The assessment was conducted on three successive sunny days at 9:00 - 12:00 and 15:00 - 17:00 in each observation stage. For each measurement, light density gradient was set to 1500, 1200, 1000, 800, 500, 200, 100, 50, 20, 0 μmol·m^-2^·s^-1^, CO_2_ was controlled constantly at 400 μmol·mol^-1^, temperature in the leaf chamber was set to 25 °C, and the air flow in the channel was set to 500 μmol·m^-2^·s^-1^. Stomatal conductance (*g*_s_), net photosynthetic rate (*P*_n_) could be directly read through Li-Cor 6400. Maximum value of photosynthesis (*P*_n-max_) and concurrent transpiration (*T*_r_) were obtained and water use efficiency (WUE) was determined according to the following equation:


(9) $$\text{WUE }=\;P_{\text{n}-\text{max}\;}\;/\;T_{\text{r}}\;\;\;\;\;\;\;\;$$

### 2.6 Statistical analysis

One-way analysis of variance (ANOVA) was used to test the effects of drought between newly sprouted and established culms in the time-series measurements. Tukey’s honest significant difference test was used to compare the means of PLC among the drought treatments. The results were considered significant at *P* < 0.05.

Linear regressions and path model analysis was conducted to explore the relationships linking relative water content of soil (RWC), percentage loss of xylem conductivity of culm (PLCC), branch (PLCB) and leaf (PLCL), stomatal conductance (*g*_s_), net photosynthetic rate (*P*_n_) and water-use efficiency (WUE).

The effects of drought-induced changes in soil moisture and the feedback of plant percentage loss of xylem conductivity of different organs,as well as plant functional leaf and hydraulic characteristics, were analyzed using structural equation modeling to understand the complex interactions between them (Vitra et al., 2019). The network of causal links among all observed variables was built with the ‘lavaan’ package of R 3.6.2.

All analyses were performed with SPSS (version 20.0, SPSS Inc., Chicago, IL, USA), origin and R 3.6.2.

## 3 Results

### 3.1 Soil moisture in the study sites in 2019

**Figure 1:**
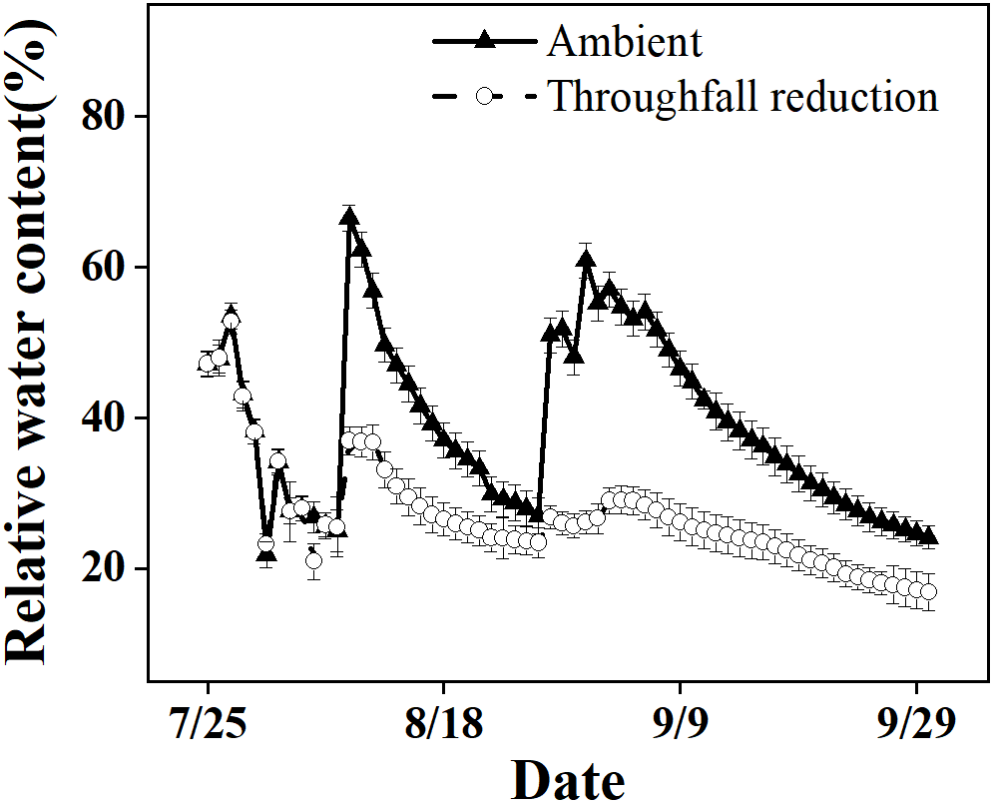
The relative soil water content in the top 20 cm layer of the ambient and throughfall reduction treatment plots in 2019.

In our study, we observed a significant reduction in RWC from 67% to 17% in the ambient plot over the course of the study period (**Figure 1**). This reduction occurred during the onset of a local summer drought, which was characterized by high temperatures and minimal rainfall. Both the ambient plot and the drought manipulated plot of Moso bamboo were exposed to these conditions, but the latter experienced greater drought intensity due to lower soil moisture levels.

### 3.2 The percentage loss of xylem hydraulic conductivity

**Figure 2:**
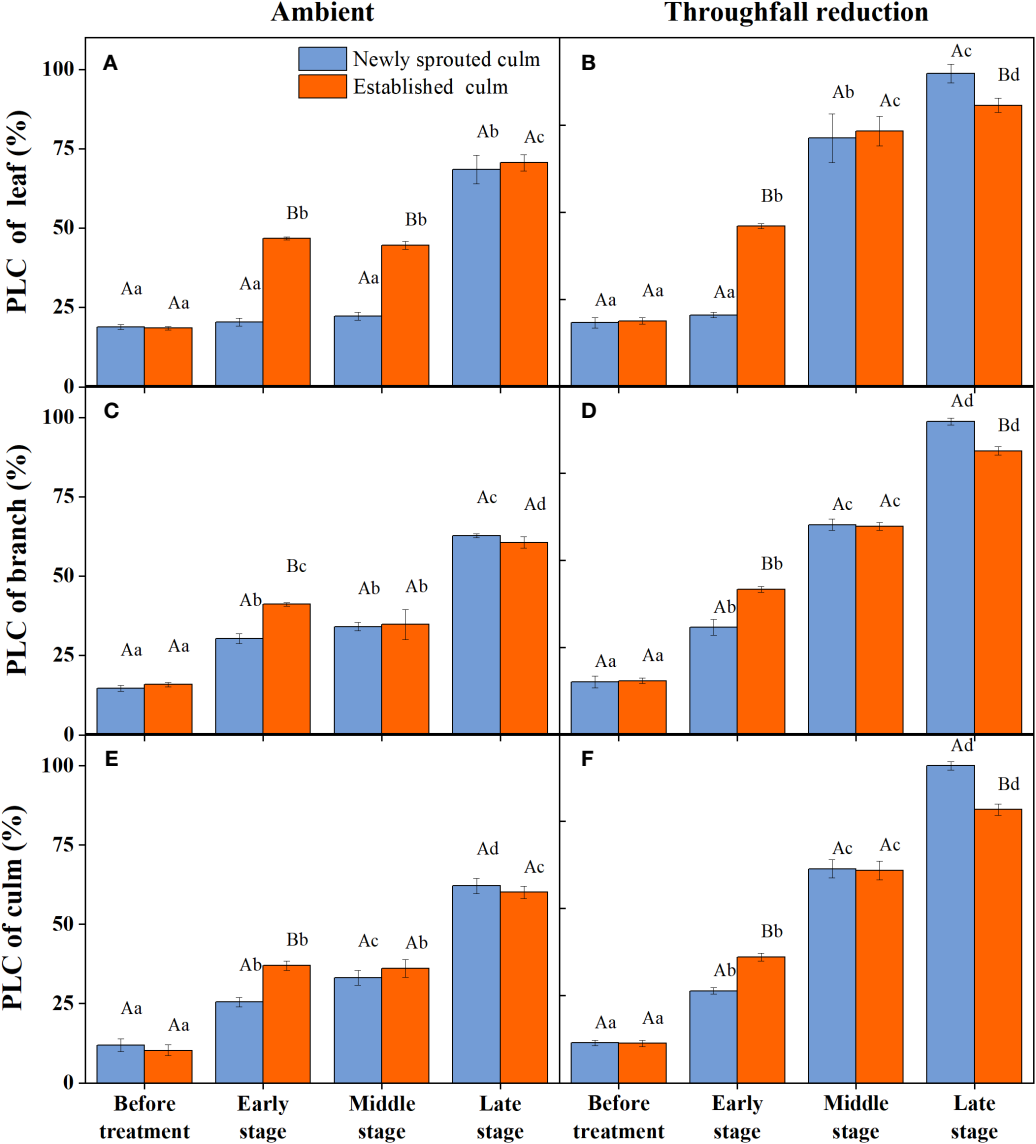
The percentage loss of xylem conductivity (PLC) of the leaf **(A, B)**, branch **(C, D)**, and culm **(E, F)** of Moso bamboo in the ambient **(A, C, E)** and throughfall reduction treatment **(B, D, F)** plots over the experimental period. Values are means ± SE (n = 3). Different uppercase letters indicate significant differences in PLC between newly sprouted culm and established culm of Moso bamboo (*P<* 0.05) in each experimental stage. For one type of culm (newly sprouted culm or established culm), different lowercase letters indicate significant differences in PLC among the experimental stages (*P*< 0.05).

**Figure 3:**
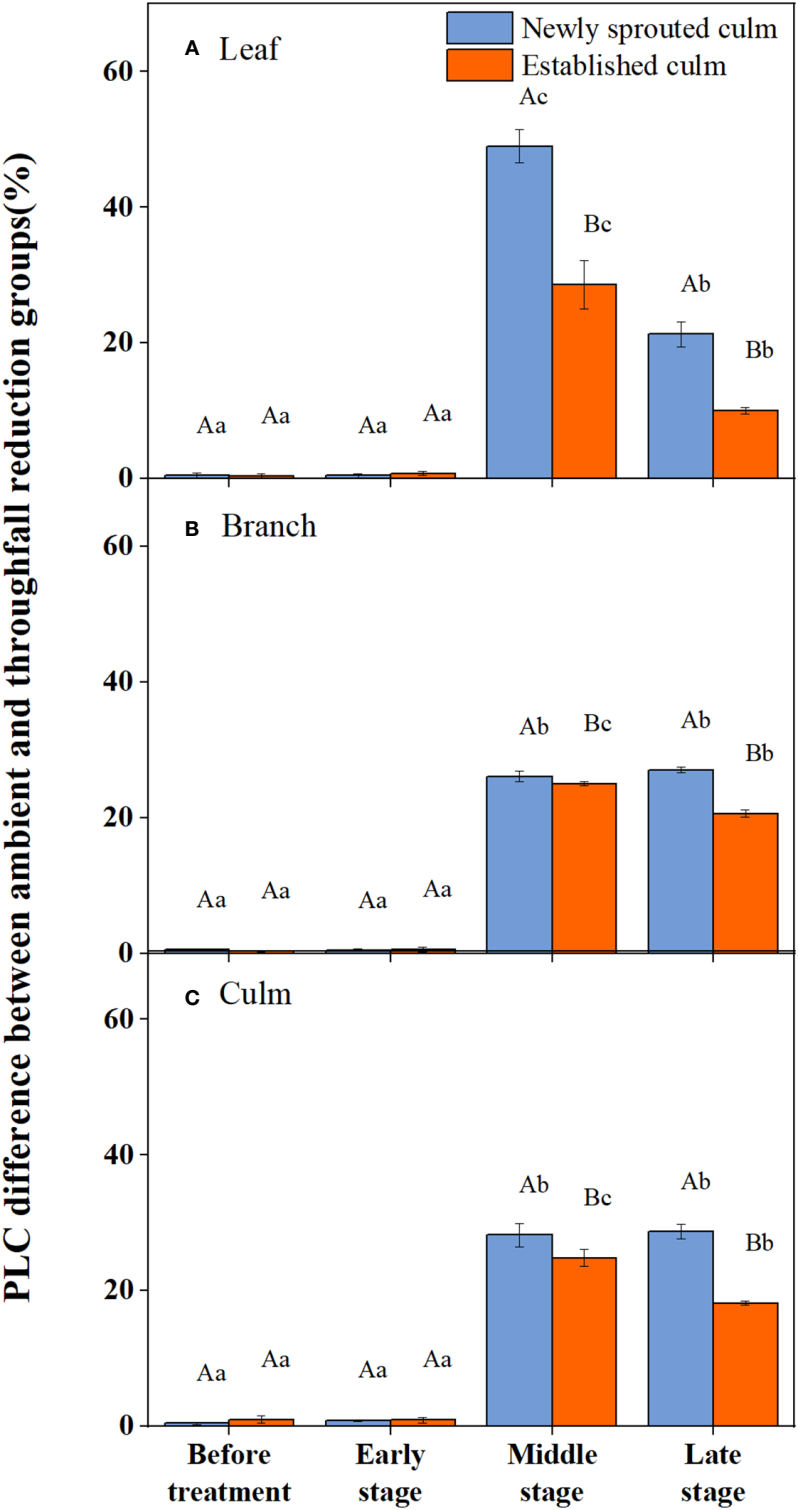
The percentage loss of xylem conductivity (PLC) difference of the leaf **(A)**, branch **(B)**, culm **(C)** of Moso bamboo subjected to drought treatment between ambient and throughfall reduction groups.Values are means ± SE (n = 3). Different uppercase letters indicate significant differences in PLC between newly sprouted culm and established culm of Moso bamboo (*P<* 0.05) in each experimental stage. For one type of culm (newly sprouted culm or established culm), different lowercase letters indicate significant differences in PLC among the experimental stages (*P*< 0.05).

**Figure 4:**
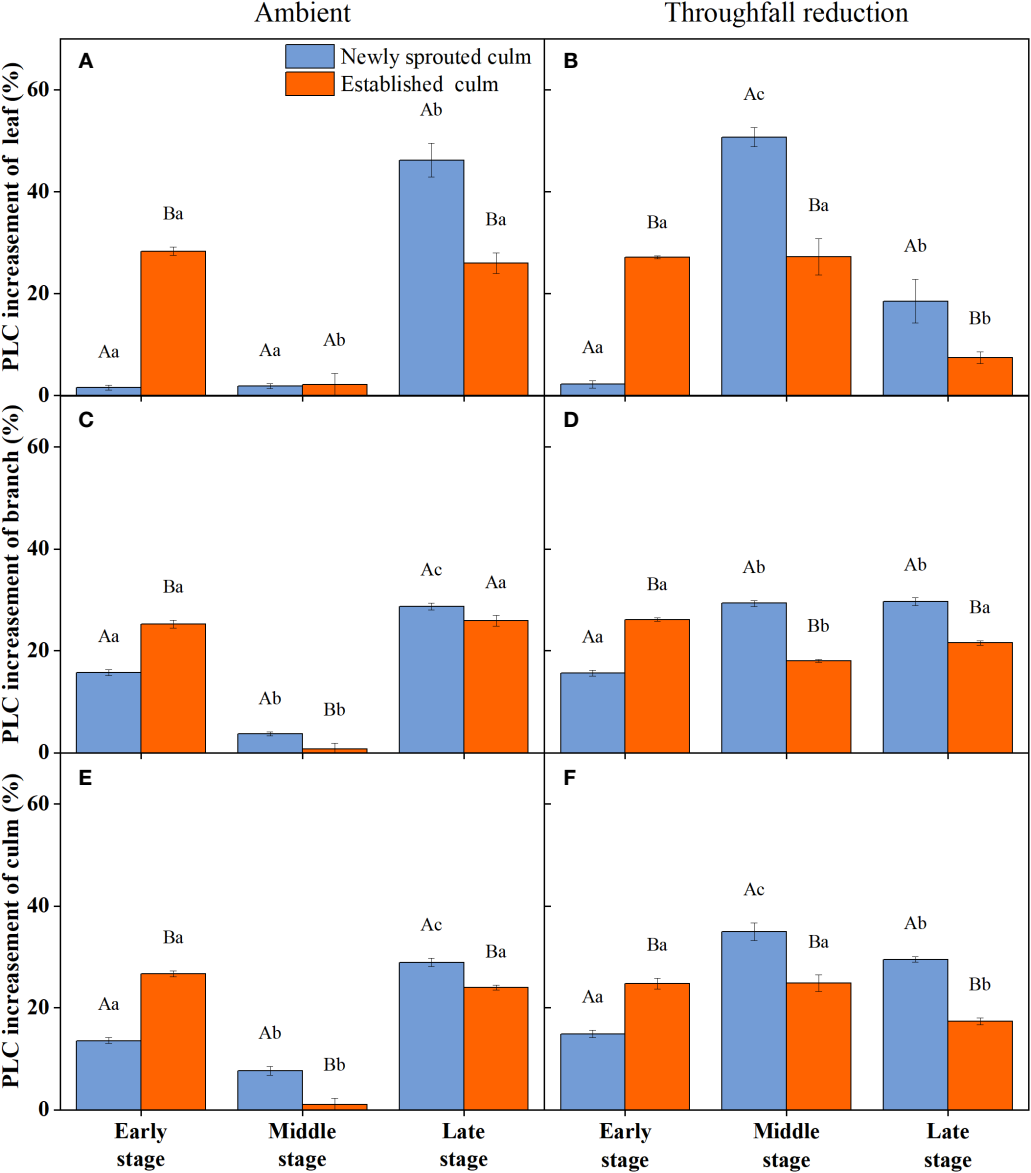
The increasement of percentage loss of xylem conductivity (PLC) of the leaf **(A, B)**, branch **(C, D)**, and culm **(E, F)** of Moso bamboo in the ambient **(A, C, E)** and throughfall reduction treatment **(B, D, F)** plots between two adjacent stages of the experiment. Values are means ± SE (n = 3). Different uppercase letters indicate significant differences in PLC between newly sprouted culm and established culm of Moso bamboo (*P<* 0.05) in each experimental stage. For one type of culm (newly sprouted culm or established culm), different lowercase letters indicate significant differences in PLC among the experimental stages (*P*< 0.05).

During the experiment, the percentage loss of xylem hydraulic conductivity (PLC) in different organs was observed between connected newly sprouted and established individuals of Moso bamboo. These observations revealed differences between the two environments (**Figure 2**). As the drought conditions persisted, the PLC of different developmental stages increased significantly in both the ambient and throughfall reduction groups. During the middle and late stages of the drought, the throughfall reduction group showed a significantly greater increase in PLC compared to the control group (*P*<0.05). Furthermore, significant differences (*P*<0.05) were observed between the newly sprouted bamboo and the established individuals (**Figures 2**, **3**, **Table 1**). The effects of both drought and age were noteworthy, and changes to various extents were observed in different organs of Moso bamboo. At the onset of the drought treatment, the increasement of PLC in newly sprouted culms was significantly lower than that of the established culms (*P*<0.05). However, with the intensification of drought conditions, the increasement of PLC in newly sprouted bamboo gradually surpassed that of established culms in the middle and end of the throughfall reduction treatment (**Figure 4**). The difference in the increasement of PLC between newly sprouted and established bamboo was greater in the throughfall reduction plot than in the ambient plot. Additionally, the difference was greater for leaves than for branches or culms (**Figure 4**). In a word, the PLC in different organs were affected to varying degrees by three factors: drought stage, treatment and stem age. The variation in PLC was primarily influenced by the drought stage, with the impact of age decreasing sequentially for PLCL, PLCB, and PLCC (*P*<0.05). Notably, age had a significant impact at the leaf level (**Table 2**).

**Table 1:** The difference in PLC of each organ (leaf, branch, and culm) between the ambient and throughfall reduction plots in each experimental stage (Before treatment, Early, Middle and Late stages).

Organ	Experimental stage	Age	PLC in Ambient Plot	PLC in Throughfall reduction plot	Significance
Leaf	Before treatment	Newly	0.189± 0.008	0.184± 0.015	–
		Established	0.185± 0.006	0.189± 0.009	–
	Early stage	Newly	0.204± 0.013	0.206± 0.008	–
		Established	0.468± 0.004	0.460± 0.007	–
	Middle stage	Newly	0.223± 0.012	0.713± 0.069	**
		Established	0.446± 0.012	0.732± 0.042	**
	Late stage	Newly	0.684± 0.045	0.898± 0.026	**
		Established	0.706± 0.026	0.806± 0.021	**
Branch	Before treatment	Newly	0.146± 0.009	0.152± 0.017	–
		Established	0.159± 0.007	0.155± 0.008	–
	Early stage	Newly	0.303± 0.015	0.308± 0.023	–
		Established	0.411± 0.005	0.417± 0.009	–
	Middle stage	Newly	0.341± 0.013	0.602± 0.017	**
		Established	0.347± 0.047	0.597± 0.012	**
	Late stage	Newly	0.628± 0.006	0.898± 0.010	**
		Established	0.606± 0.017	0.813± 0.012	**
Culm	Before treatment	Newly	0.119± 0.020	0.115± 0.008	–
		Established	0.103± 0.017	0.113± 0.009	–
	Early stage	Newly	0.255± 0.015	0.263± 0.009	–
		Established	0.369± 0.015	0.360± 0.011	–
	Middle stage	Newly	0.331± 0.023	0.613± 0.026	**
		Established	0.361± 0.028	0.609± 0.027	**
	Late stage	Newly	0.620± 0.023	0.908± 0.013	**
		Established	0.601± 0.019	0.782± 0.016	**

Double asterisks indicate a significant difference (*P*< 0.05).

**Table 2:** Three-way ANOVA (drought stage, treatment and stem age) results for concentration of PLC of each organ (leaf, branch, and culm).

Organ	Factors	Partial η^2^	df	F	P
PLCL	Drought stage	0.985	3	720.897	**<0.01**
	Treatment (control/throughfall)	0.865	1	204.498	**<0.01**
	Stem age	0.723	1	83.616	**<0.01**
PLCB	Drought stage	0.993	3	1574.055	**<0.01**
	Treatment (control/throughfall)	0.929	1	417.053	**<0.01**
	Stem age	0.175	1	6.790	0.014
PLCC	Drought stage	0.993	3	1575.513	**<0.01**
	Treatment (control/throughfall)	0.919	1	365.270	**<0.01**
	Stem age	0.057	1	1.935	0.174

Bold numbers indicate a significant effect (*P*< 0.05).

### 3.3 Leaf water potential, photosynthesis and stomatal conductance

**Figure 5:**
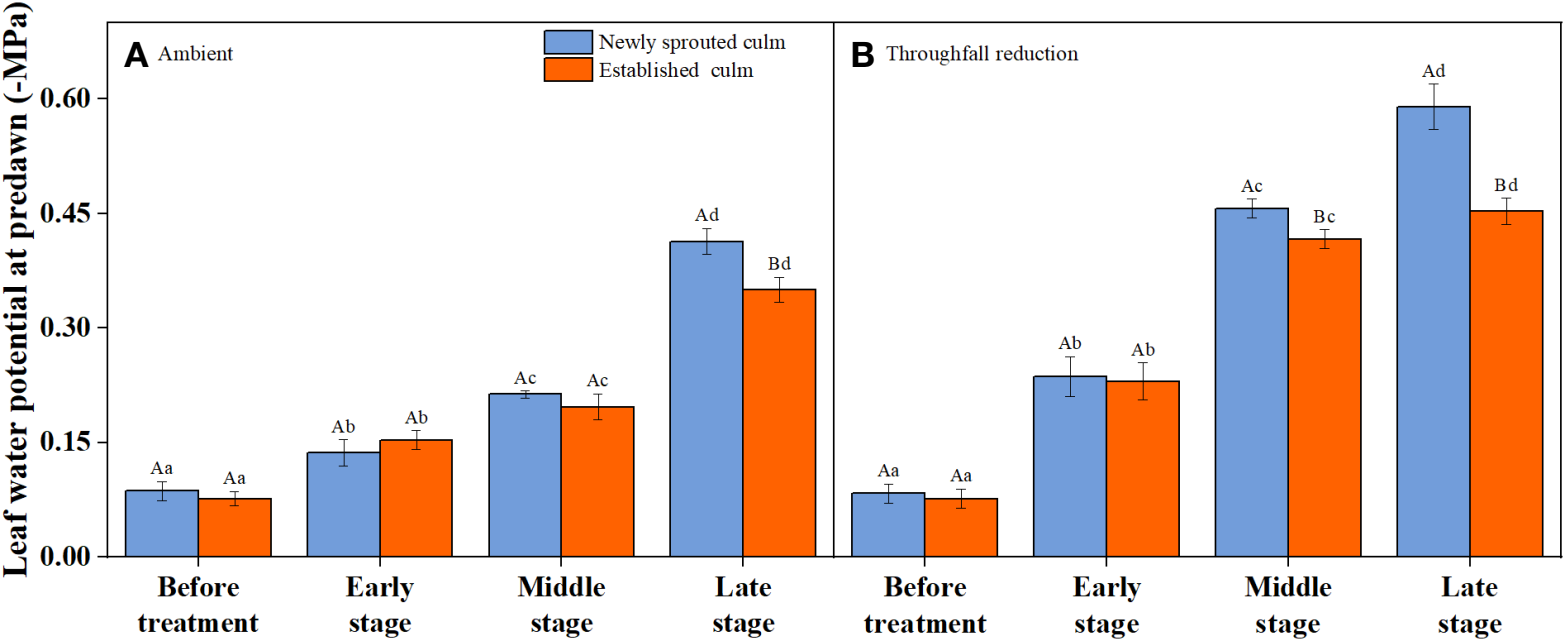
The leaf water potential at predawn (*Ψ_predawn_*) of Moso bamboo subjected to drought treatment between ambient **(A)** and throughfall reduction **(B)** groups. Values are means ± SE (n = 3). Different uppercase letters indicate significant differences in PLC between newly sprouted culm and established culm of Moso bamboo (*P*< 0.05) in each experimental stage. For one type of culm (newly sprouted culm or established culm), different lowercase letters indicate significant differences in PLC among the experimental stages (*P*< 0.05).

It is obvious that the leaf water potential at predawn (*Ψ_predawn_*) shows a significant decrease (*P*<0.05) as the drought stage progresses, whether in the control group or the throughfall interception group (**Figure 5**). Variation of *Ψ_predawn_* was mainly influenced by drought stage (**Table 3**). During the late stage of the drought, there was a significant difference (*P*<0.05) between the predawn water potential of newly sprouted and established Moso bamboo (**Figure 5**).

**Table 3:** Three-way ANOVA (drought stage, treatment and stem age) results for concentration of physiological traits of leaves.

Physiological traits of leaves	Factors	Partial η^2^	df	F	P
*Ψ_predawn_*	Drought stage	0.985	3	715.877	**<0.01**
	Treatment (control/throughfall)	0.919	1	363.582	**<0.01**
	Stem age	0.484	1	30.005	**<0.01**
*g*_s_	Drought stage	0.993	3	1427.476	**<0.01**
	Treatment (control/throughfall)	0.859	1	194.207	**<0.01**
	Stem age	0.876	1	225.078	**<0.01**
*P*_n_	Drought stage	0.991	3	1194.219	**<0.01**
	Treatment (control/throughfall)	0.906	1	308.690	**<0.01**
	Stem age	0.591	1	46.264	**<0.01**
WUE	Drought stage	0.979	3	503.690	**<0.01**
	Treatment (control/throughfall)	0.801	1	128.504	**<0.01**
	Stem age	0.619	1	51.990	**<0.01**

Bold numbers indicate a significant effect (*P*< 0.05).

**Figure 6:**
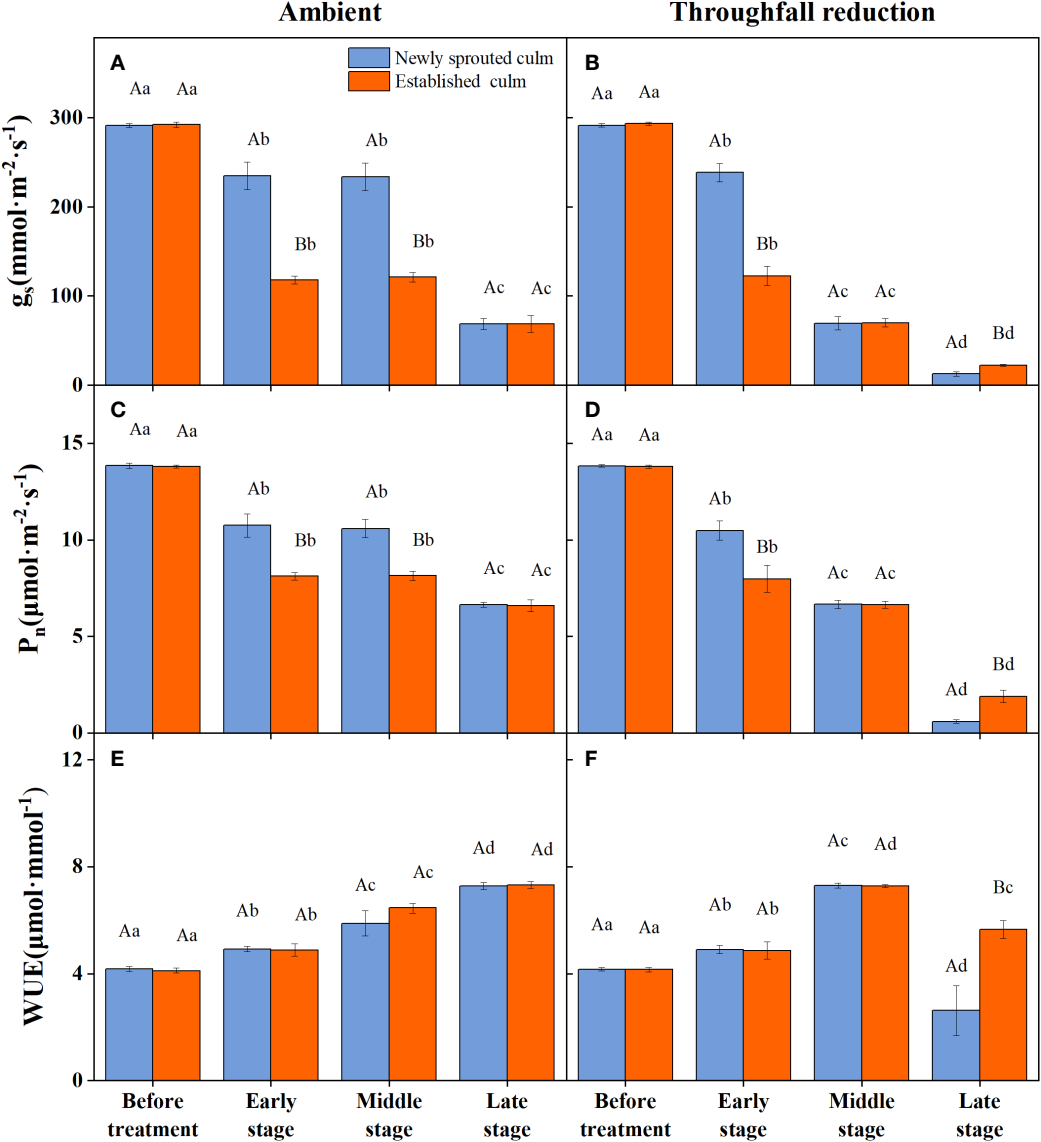
The stomatal conductance (*gs*, **A, B**), photosynthetic rate (*Pn*, **C, D**) and water use efficiency (WUE, **E, F**) of the leaf of Moso bamboo in the ambient **(A, C, E)** and throughfall reduction treatment **(B, D, F)** plots. Values are means ± SE (n = 3). Different uppercase letters indicate significant differences in PLC between newly sprouted culm and established culm of Moso bamboo (*P*< 0.05) in each experimental stage. For one type of culm (newly sprouted culm or established culm), different lowercase letters indicate significant differences in PLC among the experimental stages (*P*< 0.05).

The coupling between CO_2_ and water gas exchange in the leaf, and the similar leaf physiology among clonal species, yielded similar patterns but obviously different values of connected newly sprouted and established culm of Moso bamboo between photosynthesis and stomatal conductance (**Figures 6A–D**). During drought, the photosynthetic rate (*P*_n_) of both age-groups decreased substantially, with a significant variation (*P*<0.05) in each stage of experiments (**Figures 6C, D**, **Table 4**). The trends of the *g*_s_ in Moso bamboo were also similar, and both were significantly impacted (*P*<0.05) by drought and age (**Figures 6A, B**, **Table 4**). Intrinsic water-use efficiency (WUE) was nearly two-fold higher in the established *vs*. the newly sprouted culms at the ending stage of severe drought (**Figure 6F**, **Table 4**). This large difference was driven by the lower photosynthetic rate and stomatal conductance of the newly sprouted culms compared to the established during the late stage, as a response to the extremely low relative humidity in the throughfall reduction site (**Figure 6**, **Table 4**). It was apparent that the stomatal conductance, photosynthetic rate and water-use efficiency were also significantly influenced (*P*<0.05) by three factors: drought stage, treatment and stem age (**Table 3**).

**Table 4:** The difference in the stomatal conductance (*g_s_*), photosynthetic rate (*P_n_*) and water use efficiency (WUE) of the leaf between the ambient and throughfall reduction plots in each experimental stage (Before treatment, Early, Middle and Late stages).

Physiological characteristics of leaves	Experimental stage	Age	Numerical value in Ambient Plot	Numerical value in Throughfall reduction plot	Significance
*g_s_* (mmol·m^-2^·s^-1^)	Before treatment	Newly	291± 2.06	292± 2.02	–
		Established	292± 3.21	293± 1.81	–
	Early stage	Newly	235± 15.4	239± 10.2	–
		Established	118± 4.42	123± 10.9	–
	Middle stage	Newly	234± 15.5	69.5± 7.32	**
		Established	121± 5.17	69.9± 4.73	**
	Late stage	Newly	68.8± 6.26	12.7± 2.67	**
		Established	68.8± 9.42	22.3± 1.12	**
*P_n_* (μmol·m^-2^·s^-1^)	Before treatment	Newly	13.8± 0.142	13.8± 0.073	–
		Established	13.8± 0.079	13.8± 0.089	–
	Early stage	Newly	10.7± 0.604	10.5± 0.504	–
		Established	8.11± 0.199	7.98± 0.703	–
	Middle stage	Newly	10.6± 0.462	6.66± 0.221	**
		Established	8.14± 0.251	6.63± 0.180	**
	Late stage	Newly	6.63± 0.130	0.594± 0.095	**
		Established	6.58± 0.311	1.90± 0.322	**
WUE (μmol·mmol^-1^)	Before treatment	Newly	4.17± 0.012	4.17± 0.069	–
		Established	4.11± 0.011	4.16± 0.081	–
	Early stage	Newly	4.92± 0.113	4.90± 0.152	–
		Established	4.88± 0.234	4.86± 0.319	–
	Middle stage	Newly	5.88± 0.471	7.30± 0.093	**
		Established	6.45± 0.178	7.28± 0.062	**
	Late stage	Newly	7.28± 0.124	2.62± 0.937	**
		Established	7.31± 0.133	5.65± 0.332	**

Double asterisks indicate a significant difference (*P*< 0.05).

### 3.4 Interaction effects of plant hydraulic and physiological traits responses to summer drought

**Figure 7:**
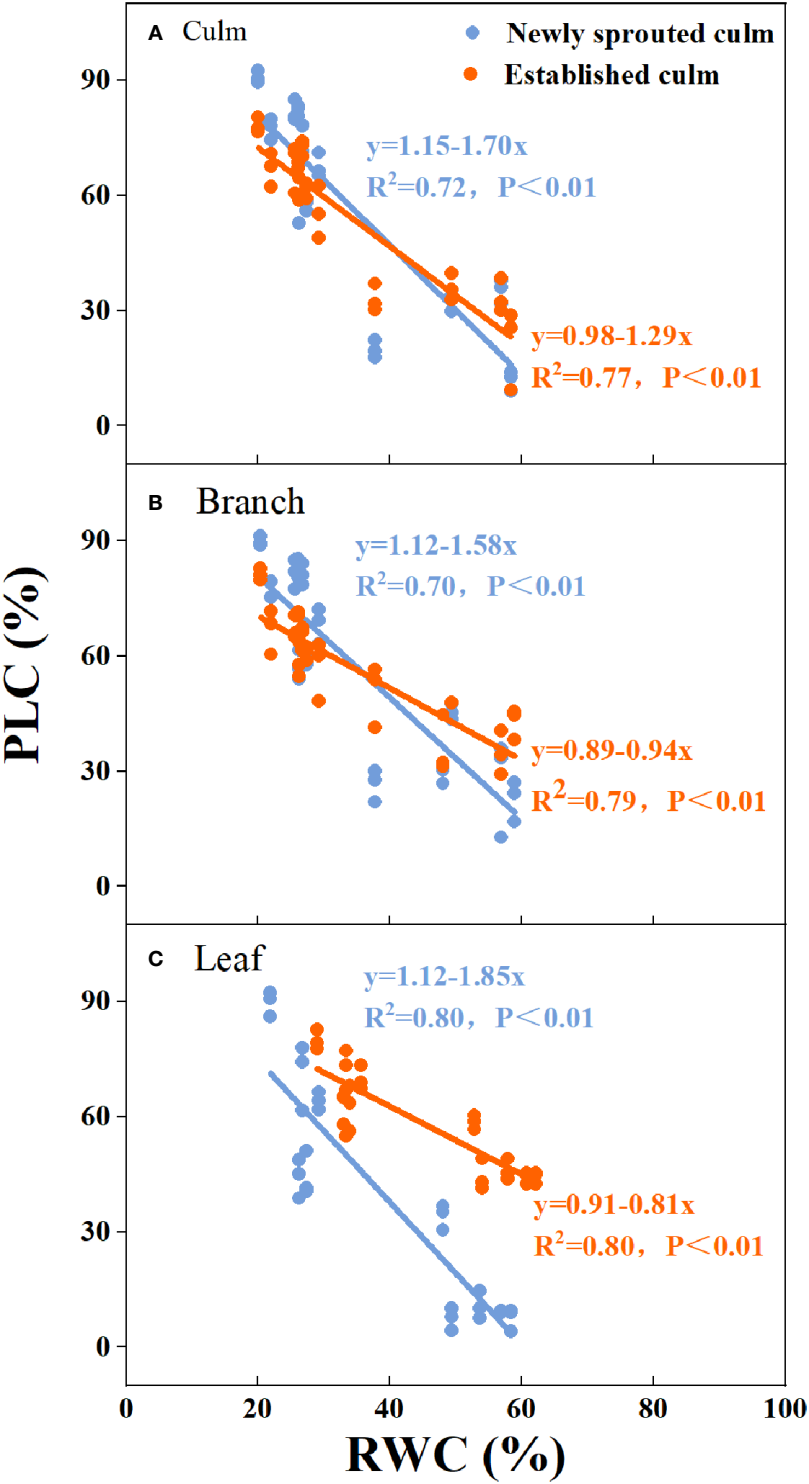
The relationship between relative water content of soil (RWC) versus percentage loss of xylem conductivity (PLC) for the culm **(A)**, branch **(B)**, and leaf **(C)** of Moso bamboo between newly sprouted and established culms.

**Figure 8:**
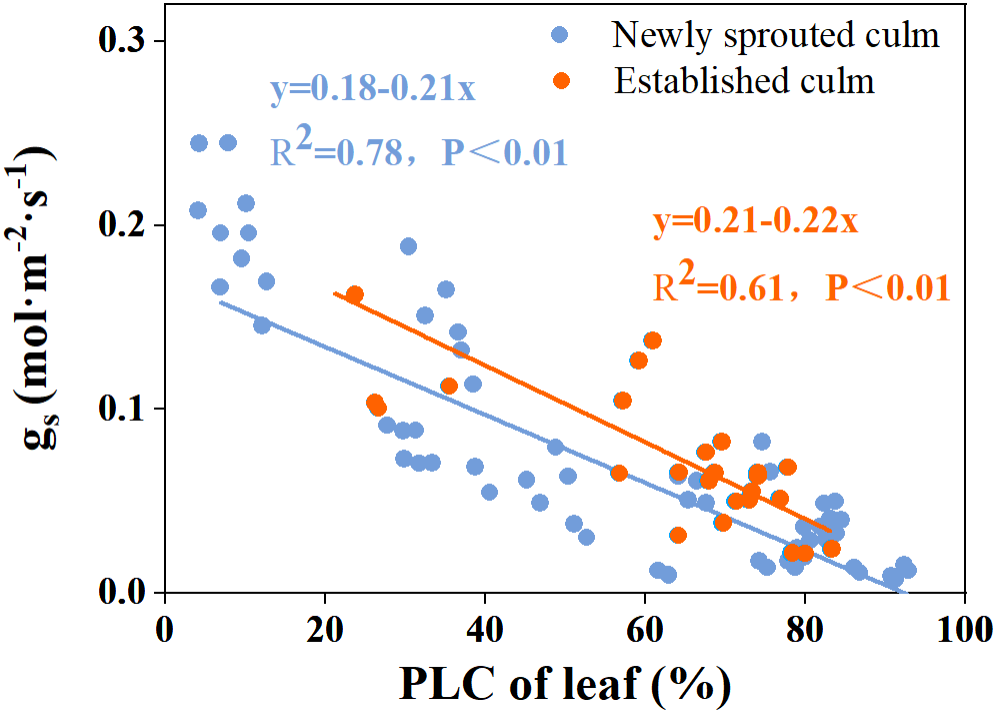
The relationship between percentage loss of xylem conductivity (PLC) versus stomatal conductance (*g*_s_) in the leaf of Moso bamboo between newly sprouted and established culms.

**Figure 9:**
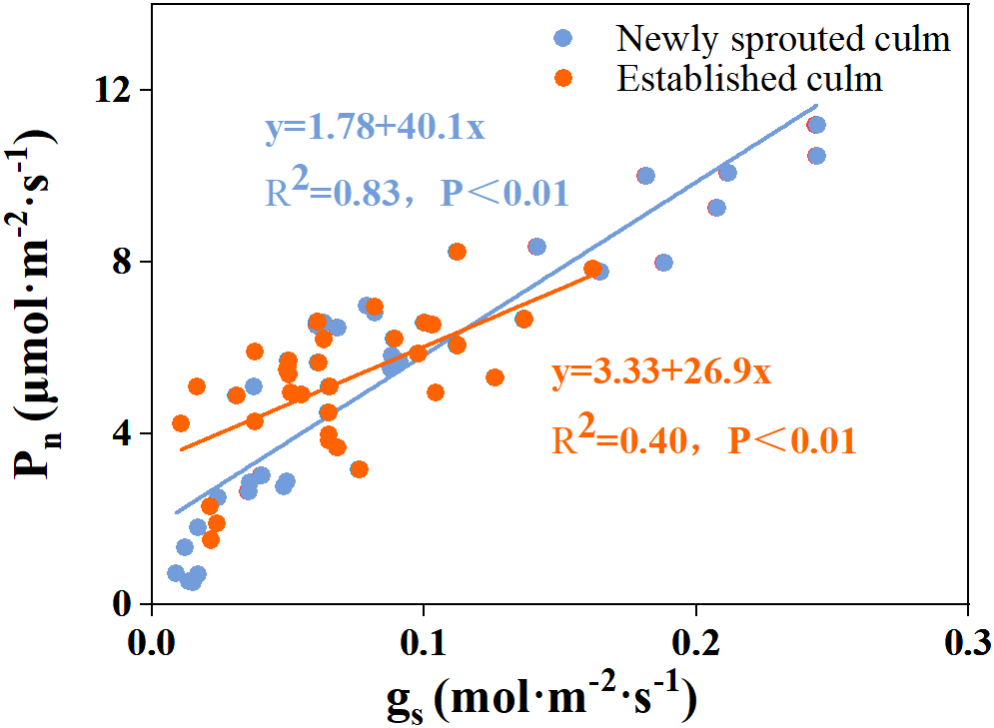
The relationship between stomatal conductance (*g*_s_) versus photosynthetic rate (*P*_n_) in the leaf of Moso bamboo between newly sprouted and established culms.

There was a negative relationship between PLC and RWC for the culm, branch, leaf of Moso bamboo between newly sprouted and established clonal groups (*R* ^2^ ≥ 0.70, *P*< 0.01), i.e., the newly sprouted culms had a more positive PLC than those of the established in low RWC (**Figure 7**). Moreover, the slope of the linear regression was significantly steeper for newly than for the established groups in different tissues (*P*< 0.05, **Figure 7**). In the leaves, the results of Pearson correlation showed that *g*_s_ was significantly inversely correlated with PLC, and was positively correlated with *P*_n_ (**Figures 8**, **9**). The Moso bamboo of different age with greater gs tended to have lower PLC and higher *P*_n_, which confirms that *g*_s_ was a key link between hydraulic traits and carbon input. Of course, the slope of the linear regression had obvious difference (*P*<0.05) between the newly and established clonal groups of Moso bamboo (**Figures 8**, **9**).

## 4 Discussion

### 4.1 The percentage loss of xylem hydraulic conductivity to summer drought

As anticipated, drought had notable impacts on PLC, which could lead to hydraulic failure and death of the plant (Blackman et al., 2009; McDowell, 2011). In this investigation, the Moso bamboo individuals encountered inadequate soil moisture that was enough to trigger xylem embolism, potentially threatening plant survival due to PLC surpassing the threshold of 85% (**Figure 2**). It seemed that most gymnosperm trees demonstrated fatal hydraulic failure at a 50% decline in conductivity, whereas angiosperms normally reached this stage later (88 or 90% loss of conductivity) (Urli et al., 2013; Li et al., 2016). The PLC of Moso bamboo exhibited varying degrees of changes across different organs under the influence of drought, consistent with previous studies (Adams et al., 2009; Yan et al., 2017). This illustrated that the hydraulic response of plants to drought was a holistic response rather than functional changes of a single organ. In this study, the PLC in different organs were significantly influenced (*P*<0.05) by the duration and intensity of drought (**Table 2**), indicating that water transport system of Moso bamboo is vulnerable to drought stress. As in previous studies, the sap flow density of Moso bamboo culms was remarkably declined under manipulated drought (Wu et al., 2019; Tong et al., 2021), and mean daily transpiration was descended to 80% during summer drought(Gu et al., 2019). Furthermore, it seemed that the PLC differed among organs at least under ambient condition (**Figure 2**). Hence, hydraulic segmentation confirmed in some studies (Levionnois et al., 2020; Li et al., 2023) might exist between leaves and branches of Moso bamboo.

In this study, the age of Moso bamboo showed remarkable effects on the hydraulic traits during drought, which is consistent with previous studies (Aakala et al., 2013; Jiao et al., 2020). The new culm of Moso bamboo had higher sap flux densities in both ambient and throughfall reduction plots (Tong et al., 2021), as well as lower hydraulic conductance of leaf (Wu et al., 2023). During the late stage of the drought, there was a significant difference (*P*<0.05) between PLC of newly sprouted and established culms (**Figure 2**; **Table 2**). The PLC of newly sprouted culms was closer to the plant hydraulic failure death threshold. It indicated that culm age has a significant impact on the water transport system under extreme drought conditions, and the new one was more prone to death under severe drought.

### 4.2 Physiological traits of leaf to summer drought

The leaf *Ψ_predawn_, g*_s_, *P*_n_, and WUE of Moso bamboo showed significant changes as the summer drought progressed (**Figures 5**, **6**), which is in line with the findings of earlier research (Adams et al., 2009; Choat et al., 2012). Numerous studies have indicated that *g*_s_ is a crucial physiological mechanism in regulating tree-level plant transpiration, and its response is influenced by species-specific regulation of hydraulic homeostasis (Klein and Niu, 2014; Gu et al., 2017). Other studies have also found that photosynthesis is reduced mainly because of a decrease in *g*_s_, which restricts the availability of CO_2_ in the intercellular area (Xu and Baldocchi, 2003; Bhusal et al., 2018). Similarly, in our study, the leaf *g*_s_ of Moso bamboo had good linear correlation with PLC and *P*_n_ (**Figures 7**, **8**). These leaf traits eventually had a strong association with WUE and help trees survive drought. As found in previous research, the decrease in transpiration rate and maturation of Moso bamboo during drought conditions are mainly due to a reduction in *g*_s_, which has the advantage of preserving water status and preventing hydraulic failure(Song et al., 2017; Gu et al., 2019). A recent study also indicated that a tropical bamboo species called *Bambusa vulgaris* exhibited an aggressive water use strategy, which maintained a 50% level of PLC of leaf by changing stomatal conductance to prevent death (Ocheltree et al., 2020). Therefore, we speculate that the leaf stomatal conductance in Moso bamboo is always an important regulatory valve connected with other physiological traits, including PLC, *P*_n_, and WUE.

**Figure 10:**
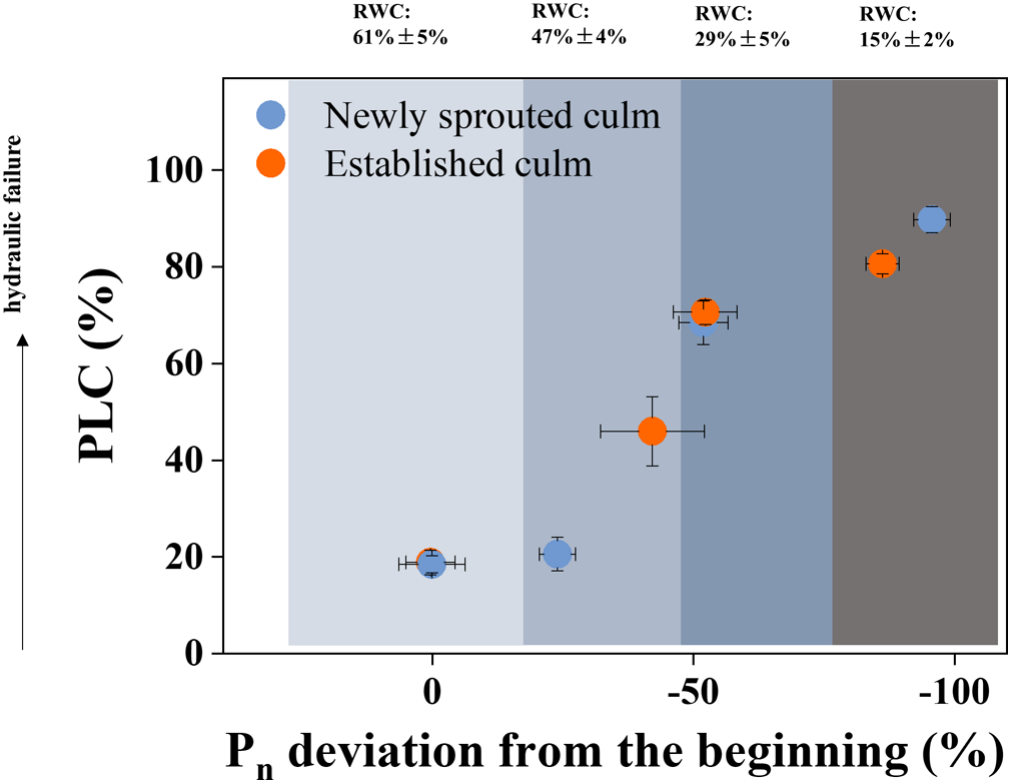
The Physiological responses associated with hydraulic trait and carbon input, as defined by the deviation of the PLC and *P*_n_ from the values of the beginning treatment. Cyan and orange symbols represent the newly sprouted and established culms, respectively.

The newly sprouted individuals had the lower PLC and higher *P*_n_ compared with those of the mature and aged groups in early drought (*P*<0.05); and with suffering severe and extreme drought, the newly Moso bamboo changed to have the higher PLC and lower *P*_n_ compared with the established individuals (*P*<0.05; **Figures 4**, **6**). To investigate the impact of summer drought conditions on hydraulic failure in Moso bamboo clonal groups, as well as the effect of age on these factors during prolonged drought, we integrated the findings concerning the PLC and *P*_n_ deviation. The outcomes indicated that as the RWC decreased, there was a rise in PLC and a decline in *P*_n_ deviation in Moso bamboo (**Figure 10**). Furthermore, the newly sprouted culms were more vulnerable of hydraulic failure than established culms in lower RWC under severe drought (**Figure 10**), which corresponds to some prior studies indicating that the newly sprouted culms of Moso bamboo had higher water use and sap flux densities (Mei et al., 2020; Tong et al., 2021). The aforementioned finding could verify a hypothesis that the newly sprouted culms were more susceptible to summer drought than established culms due to the combined effects of hydraulic damage and photosynthetic restriction, which were previously believed to be separate mechanisms (Sevanto et al., 2014; Adams et al., 2017).

### 4.3 A holistic response pattern of Moso bamboo clonal groups to summer drought

**Figure 11:**
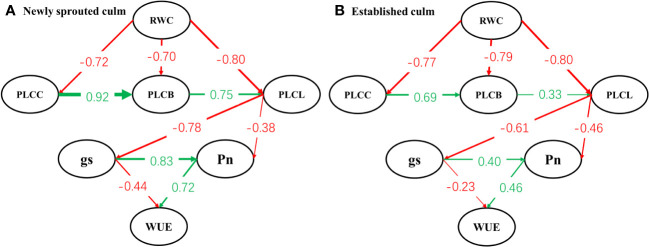
Path model analysis between newly sprouted **(A)** and established **(B)** culms of the relationships linking relative water content of soil (RWC), percentage loss of xylem conductivity of culm (PLCC), branch (PLCB) and leaf (PLCL), stomatal conductance (*g*_s_), net photosynthetic rate (*P*_n_) and water-use efficiency (WUE). Arrows shows significant relationships (pathways, *P*< 0.05) between variables, and numbers next to arrows show standardized parameter estimates.

Structural equation modelling begins with an *a priori* model which articulates the proposed causal influences expected in study network of interacting variables, based upon theory, past experience, and logic (de Vries et al., 2012). Our model (**Figure 11**) stated the general hypothesis about a holistic response pattern of Moso bamboo clonal groups to summer drought. With continuous drought, the degree of negative influence of soil moisture on hydraulic system decreases gradually along the position of organs such as stem, branch and leaf (*P*<0.05). Then the stomatal conductance, photosynthetic capacity of leaf further was influenced by the damage of hydraulic transport system through the interaction of organs. Eventually the water-use efficiency and carbon input of Moso bamboo showed a relatively large decrease under severe drought. This hypothesis also was supported by some prior research. It demonstrated that drought-induced reductions in whole-plant hydraulic conductivity further affected leaf photosynthetic capacity by limiting CO_2_ uptake via stomata, and the hydraulic and carbon processes should not be considered separately under drought stress (Dai et al., 2018; Paudel et al., 2019; Andivia et al., 2020; Chen et al., 2020).

Drought resistance is a multifactorial characteristic that involves several mechanisms, including: (i) escape by accelerating plant reproductive phase to avoid the detrimental effects of stress, (ii) avoidance by maintaining high internal water content and minimizing tissue damage, and (iii) tolerance by sustaining growth even with low internal water content over an extended drought period (Basu et al., 2016; Gupta et al., 2020). A recent investigation revealed that at each of the three developmental stages, a sympodial bamboo species (*Bambusa chungii*) adopted the tolerant strategy in response to drought (Zhang et al., 2017). By comparing the two models (**Figures 11A, B**), a core idea of this study that the newly sprouted culms were more vulnerable in the adversity and showed different effect degree but consistent response pattern(tolerant strategy)under severe drought compared to connected established culms in Moso bamboo clonal groups was proposed.

## 5 Conclusion

In this study, the response of Moso bamboo clonal groups to manipulated summer drought was studied, with a focus on the differences in hydraulic and leaf photosynthetic traits between newly sprouted and established culms. It was observed that both aged culms exhibited a similar tolerant response pattern under severe drought, but newly sprouted culms were more vulnerable to summer drought. The findings suggested hydraulic failure may largely contribute to the higher mortality of newly sprouted culms than elder culms when subjected to extreme drought. Further investigation is necessary to determine the specific function of non-structural carbohydrate reserves and clonal integration in controlling the structural and physiological characteristics of bamboo culms throughout various stages of development, which may test the mechanism of carbon starvation to the drought-induced mortality of Moso bamboo.

## Data availability statement

The original contributions presented in the study are included in the article/**Supplementary Material**. Further inquiries can be directed to the corresponding author.

## Author contributions

DF: Conceptualization, Investigation, Conducting- fieldwork, Writing- Original draft preparation, Writing- Reviewing and Editing. XZ: Methodology, Software, Investigation, Visualization, Writing- manuscript. CT: Data curation, Investigation. TM: Methodology, Conducting- fieldwork, Writing- Reviewing and Editing. YL: Conceptualization, Supervision, Funding acquisition, Writing- Reviewing and Editing. All authors contributed to the article and approved the submitted version.
