# Supplementary figures and images for: Different hydraulic and photosynthetic responses to summer drought between newly sprouted and established Moso bamboo culms
Source: Front Plant Sci. 2023 Oct 12;14:1252862. doi: 10.3389/fpls.2023.1252862 (PMC10602750; doi:10.3389/fpls.2023.1252862)

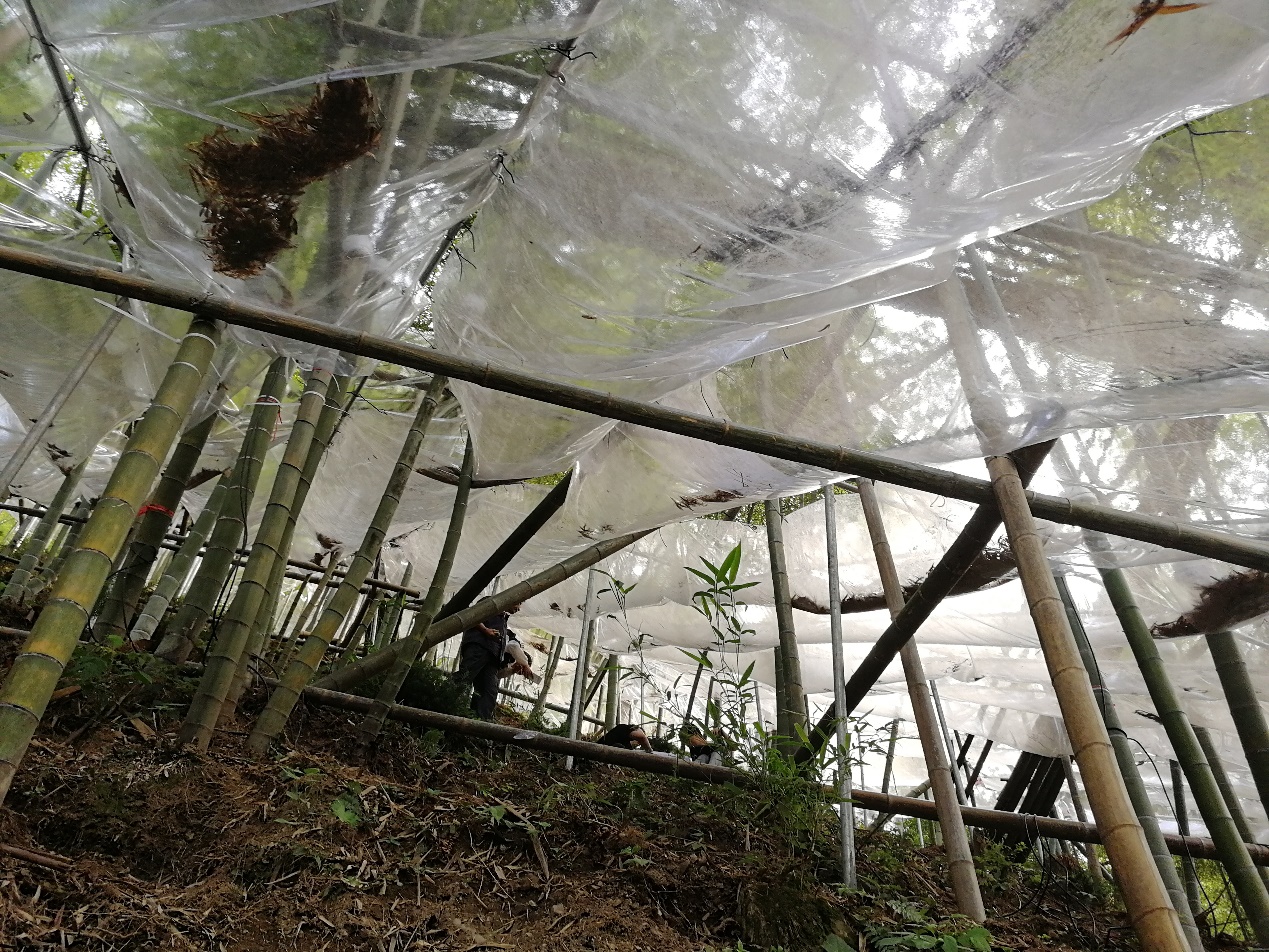


**Appendix Figure 1** The photos of the sample plot


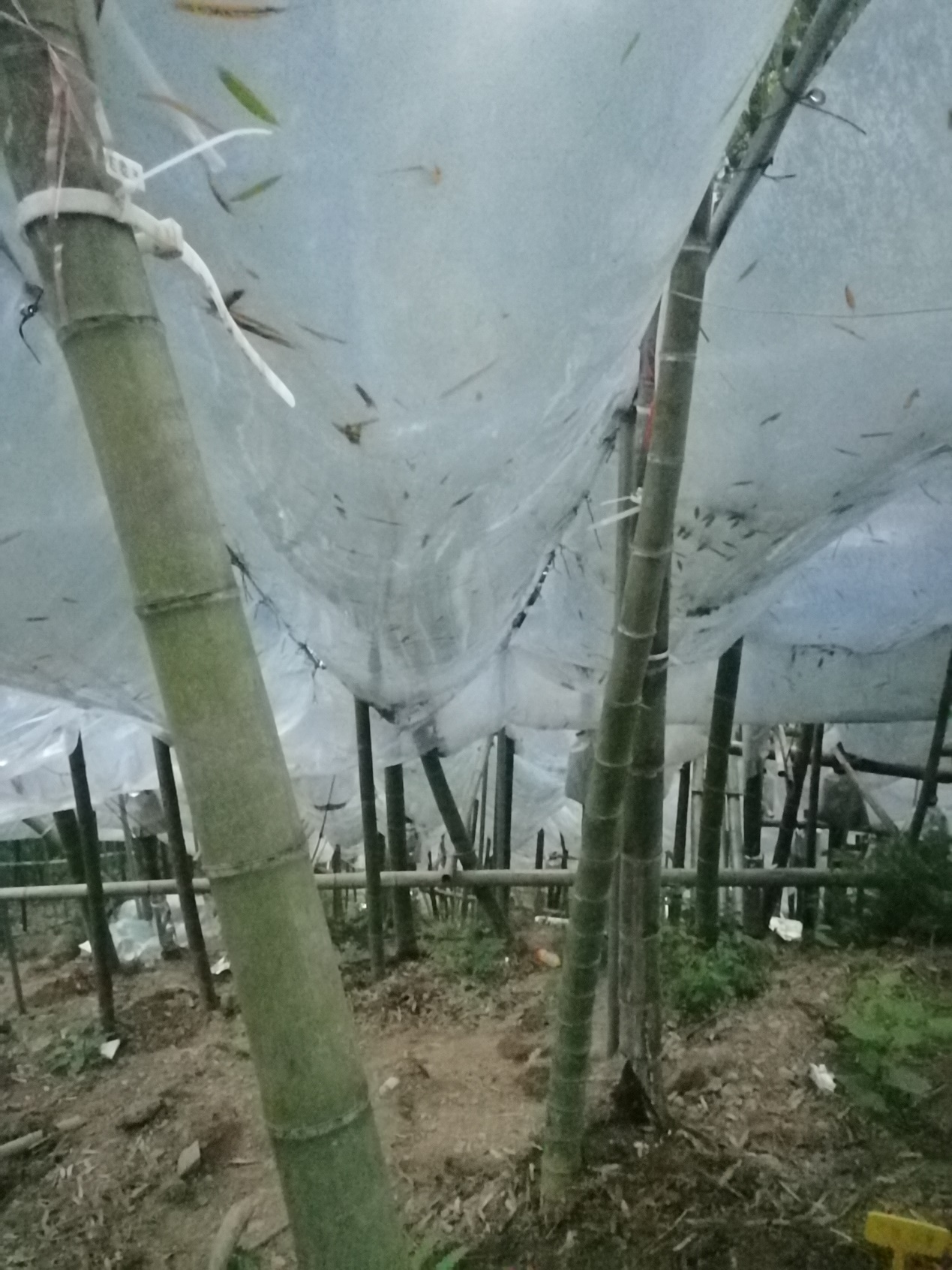


**Appendix Figure 2** The photos of the sample plot

Supplement: Supplementary file 1 [file DataSheet_1.docx]
